# Pterostilbene Activates the Nrf2-Dependent Antioxidant Response to Ameliorate Arsenic-Induced Intracellular Damage and Apoptosis in Human Keratinocytes

**DOI:** 10.3389/fphar.2019.00497

**Published:** 2019-05-08

**Authors:** Junfeng Zhou, Xinxin Ci, Xiaoyuan Ma, Qinlei Yu, Yan Cui, Yu Zhen, Shanshan Li

**Affiliations:** ^1^Department of Dermatology and Venereology, The First Hospital of Jilin University, Changchun, China; ^2^Institute of Translational Medicine, The First Hospital of Jilin University, Changchun, China; ^3^General Situation of Jilin Provincial Center for Animal Disease Control and Prevention, Jilin University, Changchun, China

**Keywords:** pterostilbene, Nrf2, arsenic, apoptosis, keratinocytes

## Abstract

The NF-E2 p45-related factor 2 (Nrf2), a transcription factor that regulates the cellular adaptive response to oxidative stress, is a target for limiting tissue damage from exposure to environmental toxins, including arsenic. In the current study, we determine whether Pterostilbene (Pts), as a potent activator of Nrf2, has a protective effect on arsenic-induced cytotoxicity and apoptosis in human keratinocytes. Human keratinocytes (HaCaT) or mouse epidermal cells (JB6) were pretreated with Pts for 24 h prior to arsenic treatment. Harvested cells were analyzed by MTT, DCFH-DA, commercial kits, Flow cytometry assay and western blot analysis. Our results demonstrated that Pts effectively regulated the viability in HaCaT and JB6 cells, decreased the reactive oxygen species (ROS) generation and lipid peroxidation (MDA), and improved the NaAsO_2_-induced depletion of superoxide dismutase (SOD). Moreover, Pts treatment further dramatically inhibited NaAsO_2_-induced apoptosis, specifically the mitochondrial mediation of apoptosis, which coincided with the effective recovery of NaAsO_2_-induced mitochondrial membrane potential (ΔΨm) depolarization and cytochrome c release from the mitochondria. Furthermore, arsenic-induced decrease of anti-apoptotic factor Bcl-2 and Bcl-xl, and increase of pro-apoptotic factor Bax and Bad, as well as survival signal related factor caspase 3 activation were reversed by Pts treatment. Further mechanistic studies confirmed that Pts increased antioxidant enzyme expression in a dose-dependent manner, which was related to Nrf2 nuclear translocation. In addition, the effects of Pts on NaAsO_2_-induced cell viability were largely weakened when Nrf2 was knocked down. Together, our results provide evidence for the use of Pts to activate the Nrf2 pathway to alleviate arsenic-induced dermal damage.

## Introduction

Arsenic, a common constituent of the Earth’s crust, affects the health and well-being of many diverse populations. Inorganic arsenic is generally considered more harmful than organic arsenic, and the most toxic arsenic compounds are in trivalent oxidation states, such as sodium arsenite (NaAsO_2_) and arsenic trioxide (As_2_O_3_). Previous studies have mainly focused on the carcinogenic effect of NaAsO_2_ and the anticancer function of As_2_O_3_ (Jiang et al., 2013). Epidemiological studies have shown that long-term exposure to NaAsO_2_ in contaminated drinking water increases the risk of many different types of cancer in the skin, lung, kidney and liver (Hughes et al., 2011; Martinez et al., 2011). Human skin lesions associated with arsenic exposure include Bowen’s disease, pigmentation disorders, squamous cell carcinoma, verrucose hyperkeratosis and basal cell carcinoma. In addition, skin complications caused by arsenic exposure occur after an incubation period of 5–10 years and continue to develop decades after the cessation of arsenic exposure, suggesting that long-term prevention is beneficial (Haque et al., 2003).

Arsenic cancer, an archetype of skin cancer, is characterized by increased proliferation, individual cell apoptosis, and full-layered dysplasia, which can be viewed microscopically. All of these effects require the involvement of mitochondria, which contribute to energy production, ROS development, cell proliferation, and DNA damage and mutations (Lee and Yu, 2016). Previous studies have shown that exposure to inorganic arsenic salts (iAs3+) may induce oxidative stress (Zhao et al., 2013). In the context of increased oxidative stress, arsenic contributes to increased oxidative damage, apoptosis and mtDNA mutations in keratinocytes and in the tumor tissues of patients with arsenic skin cancers (Lee and Yu, 2016). Nrf2, a redox-sensitive transcription factor, plays an imperative role in the cellular redox homeostasis and oxidative stress, including increasing the expression of genes encoding antioxidant and phase 2 drug-metabolizing enzymes, such as heme oxygenase1 (HO-1), NAD(P)H: quinone oxidoreductase 1 (NQO-1) and superoxide dismutase (SOD) (Kumar et al., 2014). Earlier experiments showed that the stable knockdown of endogenous Nrf2 makes the cells more sensitive to NaAsO_2_-induced cell death (Wang et al., 2007). In addition, numerous reports have also indicated that the antioxidant signaling pathway regulated by Nrf2 is involved in the adaptive response to inorganic arsenite-induced oxidative stress, and Nrf2 is considered to be a key factor in this response (Fu et al., 2010; Zhao et al., 2012). Previous studies have demonstrated that activation of Nrf2 and the Nrf2-regulated antioxidant and detoxification enzyme expression can protect HaCaT cells from arsenic-induced apoptosis and cytotoxicity (Zhao et al., 2013; Duan et al., 2016). Therefore, enhancing the Nrf2-dependent adaptive response is expected to protect against iAs (Martinez et al., 2011)^+^-induced toxicity and carcinogenicity.

To date, the study of natural products has contributed greatly to drug discovery, and many epidemiological studies have shown that phytochemicals found in fruits and vegetables can reduce the risk of developing various cancers (Koehn and Carter, 2005a,b; Gomez-Pinilla, 2008). In the past few decades, some studies have demonstrated the benefits of natural products in combating oxidative stress by regulating the Nrf2/ARE pathway (Lv et al., 2017a,b, 2018; Chen et al., 2018). Pterostilbene, a natural dimethylated analog of resveratrol, is found in blueberries and grapes. It has been reported to that Pts can prevent myocardial ischemia/reperfusion injury by reducing oxidative/nitrative stress and inflammatory responses (Yu et al., 2017). In addition, Pts protects against ischemia/reperfusion injury in the skeletal muscles via decreasing the Bax/Bcl2 ratio (Cheng et al., 2016). The B-cell lymphoma-2 (Bcl-2) family of protein, including Bcl-2, Bcl-xl, Bax, and Bad, play a key role in apoptosis (Mahieux et al., 2001). Bcl-2 and Bcl-xl are important anti-apoptotic molecules that inactivates of Bcl-2 related X protein (Bax), thereby inhibiting the intrinsic pathway of apoptosis. Bax then stimulates the release of caspases, triggering the final cascade of events and morphological changes in the process of cell apoptosis. Resveratrol has been reported to protect against arsenic trioxide-induced cardiotoxicity (Zhang et al., 2014). Pts has a longer half-life *in vivo*, and its anti-inflammatory activity and anticancer effects are stronger than those of Resv (Paul et al., 2009; Estrela et al., 2013). In addition, Pts exerts better protection than Resv against chronic UVB-induced oxidative damage (Sirerol et al., 2015). Thus, in this study, we studied the protective effects and mechanisms of Pts on arsenic-induced cytotoxicity and apoptosis in human keratinocytes. Our results suggested that the anti-apoptosis role of Pts depends on Nrf2 activation in NaAsO_2_-induced cytotoxicity in human HaCaT cells. Our findings are important not only for understanding the effects of Pts on NaAsO_2_-induced cytotoxicity in human HaCaT cells, but also for developing strategies to prevent and proper treatment of chronic arsenic poisoning, including arsenic-induced skin disorders.

## Materials and Methods

### Reagents

Pterostilbene with a purity >98% was purchased from Chengdu Pufeide Biotech Co., Ltd. (Chengdu, China). Sodium arsenite (NaAsO_2_, 99.0%) and DCFH-DA were provided by Sigma Chemical (St. Louis, MO, United States). Antibodies against Nrf2, HO-1, NQO1, Bax, Bcl-2, Bcl-xL, Caspase-3, Bad, Cytochrome c, p-AMPK, T-AMPK, p-AKT, T-AKT, Lamin B and β-actin were supplied by Cell Signaling (Boston, MA, United States) or Abcam (Cambridge, MA, United States). The MDA and SOD test kits were purchased from Nanjing Jiancheng Bioengineering Institute (Nanjing, China). The Annexin V fluorescein isothiocyanate (FITC)/Propidium Iodide (PI) apoptosis kit was purchased from BD (San Jose, CA, United States). The control siRNA and Nrf2 siRNA were purchased from Santa Cruz Biotechnology (Santa Cruz, CA, United States). Mitochondrial membrane potential assay kit with JC-1 were offered from Beyotime Institute of Biotechnology (Jiangsu, China).

### Cell Culture and Cell Treatment

The HaCaT cell, a spontaneously immortalized human epithelial cell line, was obtained from the China Cell Line Bank (Beijing, China). Cells were maintained in a full DMEM supplemented with 3 mM glutamine and 10% heat-inactivated fetal bovine serum. The mouse epidermal cell line JB6 P+ (ATCC, CRL-2010) were cultured in EMEM containing 5% heat-inactivated fetal bovine serum and 1% gentamicin. The cells were maintained in a humidified atmosphere of 5% CO_2_ at 37°.

### Cell Viability Assay

HaCaT and JB6 cells were plated in a 96-well plate at a density of 1 × 10^4^ cells/well for 24 h. To determine the preventive effect of Pts on the cell viability, cells were pretreated with Pts (7.5, 15, and 30 μM) for 1 h, and exposed to NaAsO_2_ (25 μM) for 20 h. Then, MTT solution was added to the plates and incubated for another 4 h, the formed blue formazan crystals were dissolved in DMSO. Finally, the absorbance of MTT was measured at 570 nm with a microplate reader.

### Intracellular ROS Formation Assay

Reactive oxygen species generation was measured by the DCFH-DA method. Briefly, HaCaT and JB6 cells were incubated in 96-well plates (1 × 10^4^ cells/well) for 24 h, and then the cells were treated with 25 μM NaAsO_2_ and pretreated with or without Pts (7.5, 15, and 30 μM) for 24 h. Next, the cells were incubated with serum-free medium containing DCFH-DA (10 μM, Sigma) at 37°C away from light for 2 h. The fluorescence of the dye was measured using a multi-detection reader (Bio-Tek Instruments Inc.) at excitation and emission wavelengths of 485 and 530 nm, respectively. The treated cells were visualized directly by a fluorescence microscope (Olympus, Japan) at 200× magnification.

### Intracellular MDA and SOD Assay

Intracellular lipid peroxidation (MDA) and superoxide dismutase (SOD) concentrations were measured using a commercially test kit (Nanjing Jiancheng Bioengineering Institute, Nanjing, China). HaCaT and JB6 cells were grown in 6-well plates (1 × 10^6^ cells/well) for a 24 h incubation, and then exposed to 25 mM of NaAsO_2_ and pretreated with or without Pts (7.5, 15, and 30 μM) for 24 h. At the end of the handle, cells were collected into the tubes and then lysed in ice-cold PBS by sonication followed by centrifugation at 15,000 *g* for 10 min at 4°C. The resulting supernatants were used immediately for the measurements using commercially kits.

### Apoptotic Cells Quantification

Quantification of apoptotic cells was performed using annexin V-FITC and propidium iodide (PI) double staining. HaCaT cells were seeded into 12-well plates (5 × 10^5^ cells/well) for a 24 h incubation, and then treated with 25 μM NaAsO_2_ with or without pretreatment with Pts as mentioned above. Subsequently, the cells were washed twice with ice-cold PBS, harvested by trypsin, and centrifuged at 1500 rpm/min for 5 min at 4°C. Next, cells were subjected to annexin V and PI staining, and the level of apoptosis was determined using flow cytometry (LSR II Flow Cytometer; BD Biosciences, San Jose, CA, United States).

Morphology of apoptotic cells was observed by fluorescence microscopy following DAPI staining. In brief, the treated cells were fixed with 4% polyoxymethylene for 15 min, cellular DNA was stained with 0.1% DAPI for 15 min, and then the cell morphology was immediately visualized under a fluorescence microscope (Olympus, Japan) at 200× magnification at the wavelengths of 364 nm for excitation and 454 nm for emission.

### Western Blot Analysis

HaCaT cells were plated into 6-well plates (1 × 10^6^ cells/well) for a 24 h, and then treated with 25 μM NaAsO_2_ with or without pretreatment with Pts as mentioned above. After each experiment, the cells were washed twice with cold PBS. Whole-cell protein extracts were obtained using cell lysis buffer with 1 Mm PMSF, protease inhibitors and phosphatase inhibitors. Nuclear protein and mitochondria protein extraction kits were purchased from Beyotime (Jiangsu, China). Protein concentrations were determined using the Bradford assay before storing the lysates at –80°C. Proteins were subjected to SDS-PAGE (8–12%) and then transferred to PVDF membranes and blocked by 5% non-fat milk. Immunoblotting analysis was performed using specific antibodies. Then the membranes were further incubated with HRP-conjugated secondary antibodies and detected using an ECL western blot substrate. The band intensities were quantified using ImageJ gel analysis software. The experiments were repeated three times for each experimental condition.

### JC-1 Assay for Mitochondrial Membrane Potential

HaCaT cells were plated into 12-well plate at a density of 5 × 10^5^ cells/well, treated with 25 μM NaAsO_2_ with or without pretreatment with Pts as mentioned above. Next, the cells were trypsinized and washed with PBS, then incubated with JC-1 (10 μg/ml) at 37°C in the dark for 30 min. JC-1 fluorescence was quantified through flow cytometry, in which red JC-1 aggregates were gated in the FL2 channel and green JC-1 monomers in the FL1 channel.

### Nrf2-siRNA Transfection

HaCaT cells were seeded into 6-well plate at a density of 2 × 10^5^ cells/well until the cells fusion was approximately 40–50%. Then, the siRNA transfection reagent Lipofectamine 2000 was used to transiently transfect Nrf2-siRNA or Nrf2-negative control siRNA into the cells according to the manufacturer’s protocol (Santa Cruz Biotechnology, Santa Cruz, CA). After 24 h, the transfected cells were treated with Pts for 24 h and the lysate was analyzed by Western blot.

### Statistical Analysis

All results were expressed as the means ± SEM of three independent experiments. Differences between mean values of normally distributed data were analyzed using the two-tailed Student’s *t*-test. Significance was considered at *P* < 0.05.

## Results

### Pts Inhibited NaAsO_2_-Induced Cytotoxicity in HaCaT and JB6 Cells

**FIGURE 1:**
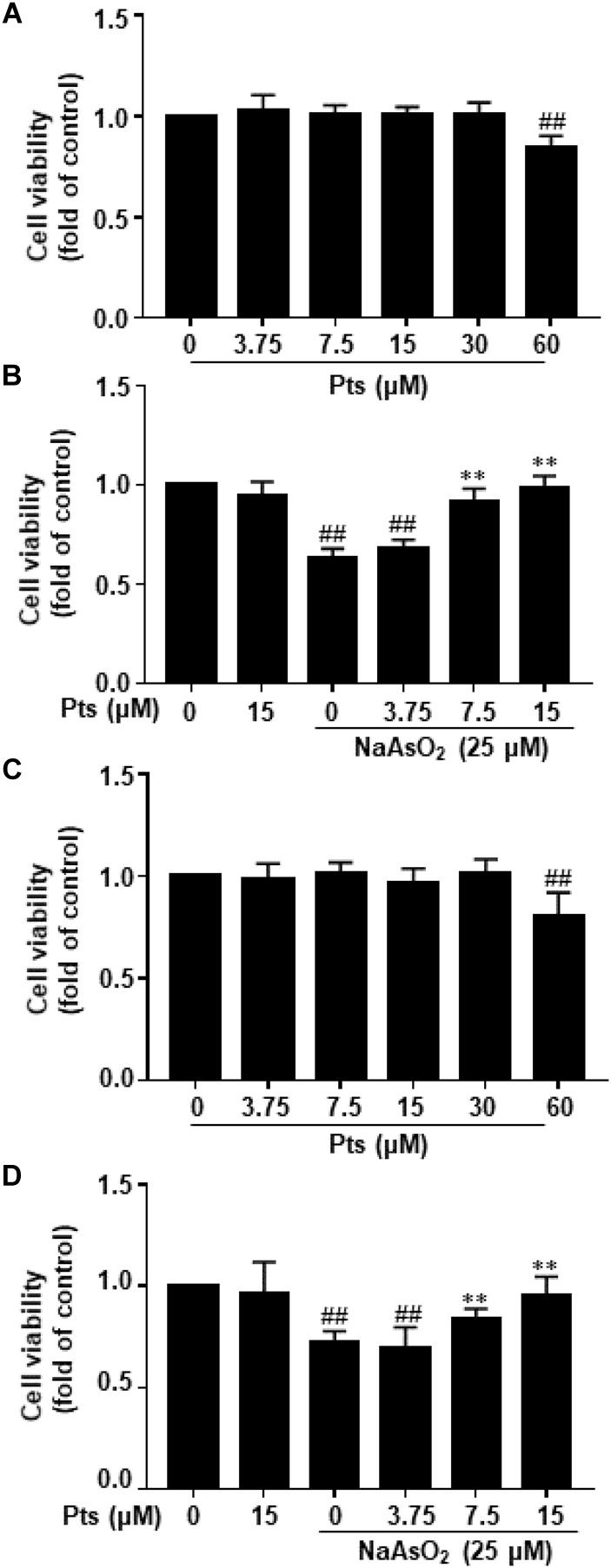
Protective effect of Pts pretreatment against NaAsO_2_-induced cytotoxicity in HaCaT and JB6 cells. **(A,C)** The HaCaT and JB6 cells were exposed to different concentrations of Pts (7.5–60 μM) for 24 h, and then cell viability was measured by MTT assay. **(B,D)** HaCaT and JB6 cells were exposed to NaAsO_2_ (25 μM) for 24 h with or without pretreatment with Pts (3.75, 7.5, and 15 μM), and then cell viability was measured by MTT assay. The results are expressed as the means ± S.E.M. of three independent experiments.^##^*P* ≤ 0.01 versus the control group, ^∗∗^*P* ≤ 0.01 versus the NaAsO_2_ group.

After cells were exposed to various concentrations of Pts for 24 h, Pts at 7.5–30 μM did not decrease the viability of HaCaT and JB6 cells compared to the control, although there was a decrease in cell viability at 60 μM (*P* ≤ 0.01, as shown in Figure 1A,C). To evaluate the protective effect of Pts against NaAsO_2_-induced cytotoxicity, Pts (7.5, 15 and 30 μM) pretreatment was conducted before NaAsO_2_ (25 μM) exposure for 24 h. Our results demonstrated that Pts remarkably protected the cells from NaAsO_2_-induced cytotoxicity in a dose-dependent manner (*P* ≤ 0.01, as shown in Figure 1B,D).

### Pts Decreased NaAsO_2_-Induced ROS Generation and MDA Formation and Increased SOD Content in HaCaT JB6 Cells

**FIGURE 2:**
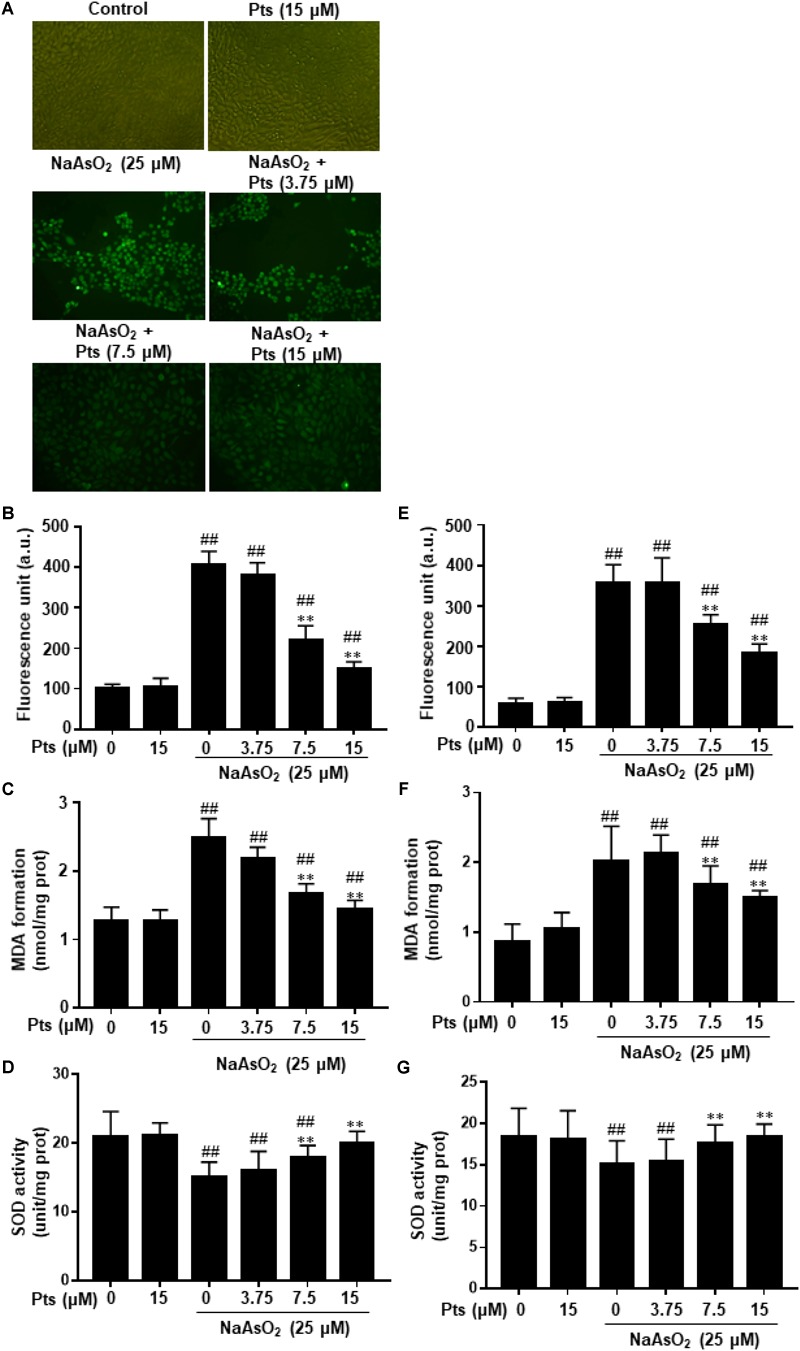
Protective effect of Pts pretreatment against NaAsO_2_-induced oxidative stress in HaCaT cells. The HaCaT and JB6 cells were exposed to 25 μM of NaAsO_2_ for 24 h with or without Pts (3.75, 7.5, and 15 μM) pretreatment. **(A,B,E)** ROS generation was measured by the DCFH-DA method using a multi-detection reader and fluorescence microscope at 100× magnification. Intracellular MDA levels **(C,F)** and SOD **(D,G)** activities were determined by commercial kits. The results are expressed as the means ± S.E.M. of three independent experiments. ^##^*P* ≤ 0.01 versus the control group, ^∗∗^*P* ≤ 0.01 versus the NaAsO_2_ group.

Increased intracellular ROS and oxidative stress induction were associated with arsenic-related cell damage (Gong et al., 2015). In this study, we found that NaAsO_2_ treatment enhanced ROS production, which was significantly inhibited by Pts pretreatment (*P* ≤ 0.01, as shown in Figure 2A,B,E). Lipid peroxidation was assessed by measuring the amounts of MDA, a common end product of lipid peroxidation. SOD is thought to be an essential antioxidant that protects against arsenic-induced oxidative damage. Figure 2C,D,F,G shows that NaAsO_2_ exposure markedly enhanced MDA generation and SOD depletion, which were reversed by Pts pretreatment (*P* ≤ 0.01).

### Pts Ameliorated NaAsO_2_-Induced Mitochondrial Dysfunction in HaCaT Cells

**FIGURE 3:**
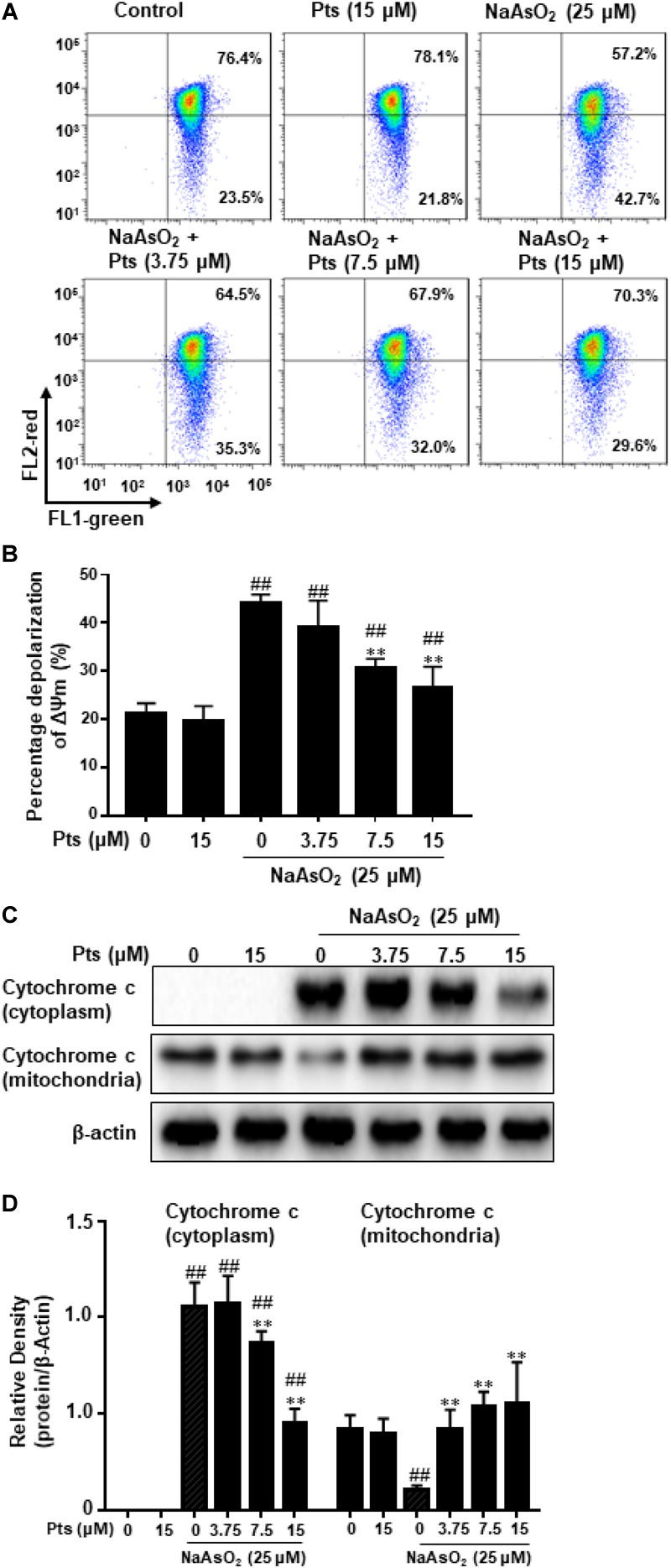
Protective effect of Pts pretreatment against NaAsO_2_-induced mitochondrial dysfunction in HaCaT cells. The HaCaT cells were exposed to 25 μM of NaAsO_2_ for 24 h with or without Pts (3.75, 7.5, and 15 μM) pretreatment. **(A)** Mitochondrial membrane potentials (ΔΨm) were assessed with JC-1 staining by flow cytometry, and the percentage of cells with depolarization of ΔΨm were quantified in panel **(B)**. **(C)** Cytochrome c levels in the mitochondria and cytoplasm were detected by western blot analysis, and the band densities were quantified in panel **(D)**. The results are expressed as the means ± S.E.M. of three independent experiments. ^##^*P* ≤ 0.01 versus the control group, ^∗∗^*P* ≤ 0.01 versus the NaAsO_2_ group.

Mitochondria, which play a vital role in the process of apoptosis in different cells, and the loss of mitochondrial membrane potential (MMP, ΔΨm) is associated with apoptosis. To determine whether NaAsO_2_-induced apoptosis in HaCaT cells occurred through a mitochondrial-derived pathway, we examined the alteration of ΔΨm and cytochrome c release in HaCaT cells. HaCaT cells were pretreated with Pts for 1 h and then exposed to NaAsO_2_ (25 μM) to induce mitochondrial dysfunction, JC-1 staining was then used to detect the change in ΔΨm. A large number of reports have showed that normal cells exhibit JC-1 red fluorescence, but the reduction of MMP caused the green JC-1 monomers to replace the red fluorescence. Compared to the control group, the ΔΨm of cells was reduced with NaAsO_2_ but was enhanced after pretreatment with Pts in a dose-dependent manner, which suggested that Pts significantly inhibited mitochondrial dysfunction in HaCaT cells (*P* ≤ 0.01, as shown in Figure 3A,B). To further determine the protective effect of Pts on mitochondria, western blot analysis was used to evaluate the release of cytochrome c from mitochondria into the cytosol. As shown in Figure 3C,D, treatment with NaAsO_2_ increased the release of cytochrome c from mitochondria into the cytosol fraction clearly, while the effect was reversed by Pts pretreatment (*P* ≤ 0.01).

### Pts Inhibited NaAsO_2_-Induced Cell Apoptosis in HaCaT Cells

**FIGURE 4:**
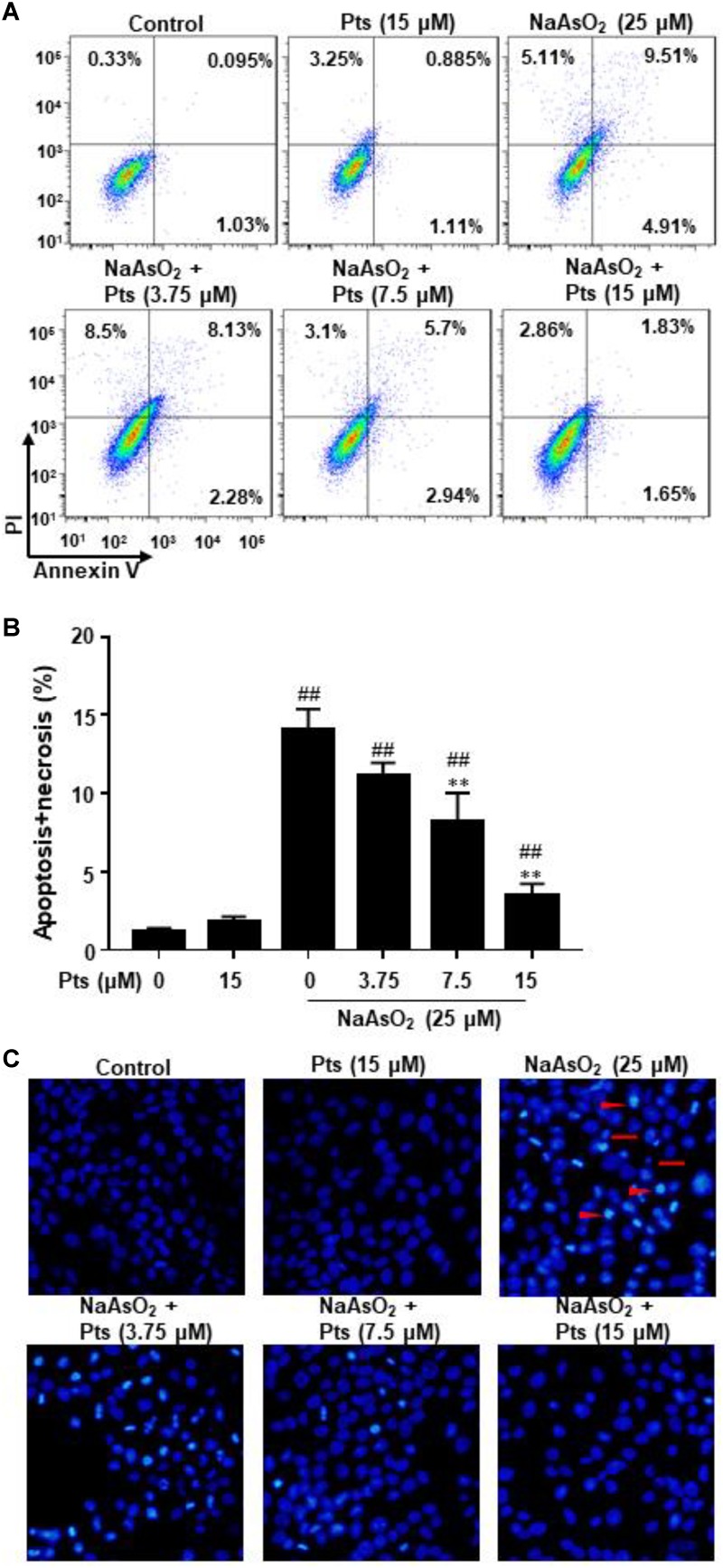
Protective effect of Pts pretreatment against NaAsO_2_-induced apoptosis in HaCaT cells. The HaCaT cells were exposed to 25 μM of NaAsO_2_ for 24 h with or without Pts (3.75, 7.5, and 15 μM) pretreatment. **(A,B)** The percentage of cell apoptosis was determined using flow cytometry. **(C)** Morphology of apoptotic cells was evaluated by fluorescence microscopy following DAPI staining at 200× magnification. Triangles: nuclear chromatin condensation in apoptotic cells. Arrows: apoptotic bodies. The results are expressed as the means ± S.E.M. of three independent experiments.^##^*P* ≤ 0.01 versus the control group, ^∗∗^*P* ≤ 0.01 versus the NaAsO_2_ group.

As shown in Figure 4A,B, NaAsO_2_ significantly increased the incidence of cell death by promoting the apoptosis and necrosis in total cells, whereas Pts effectively reduced the apoptosis and necrosis induced by NaAsO_2_ in a dose-dependent manner. We also evaluated the morphological characteristics of HaCaT cells by DAPI staining. Compared to the control group, NaAsO_2_ treatment results in typical features of apoptosis, such as chromatin condensation and apoptotic bodies, while Pts pretreatment significantly reversed those abnormalities (Figure 4C).

### Pts Modulated the Apoptosis-Related Protein Expression in Arsenic-Treated HaCaT Cells

**FIGURE 5:**
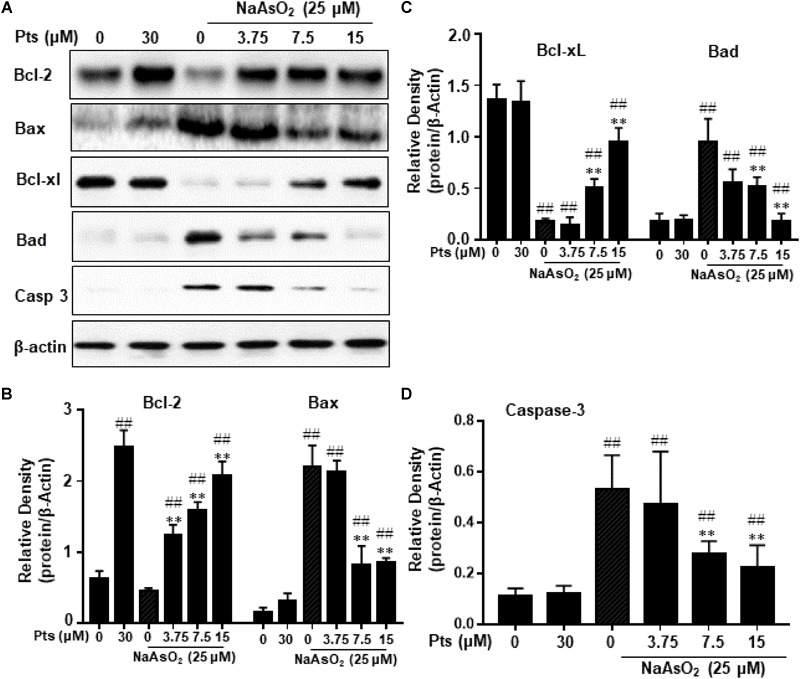
Effects of Pts on apoptosis-related protein expression in arsenic-treated HaCaT cells. The HaCaT cells were exposed to 25 μM of NaAsO_2_ for 24 h with or without Pts (3.75, 7.5, and 15 μM) pretreatment. **(A)** Bcl-2, Bax, Bcl-xl, Bad and Casp 3 protein levels from total cell lysates were detected by western blot analysis, and the band densities were quantified in panels **(B–D)**. The results are expressed as the means ± S.E.M. of three independent experiments. ^##^*P* ≤ 0.01 versus the control group, ^∗∗^*P* ≤ 0.01 versus the NaAsO_2_ group.

The Bcl-2 family of proteins, including Bcl-2, Bax, Bad, and Bcl-xl, plays an important role in apoptosis. In order to further prove the possible mechanisms of Pts in anti-apoptosis, we tested its effects on NaAsO_2_-induced apoptosis-related proteins. As shown in Figure 5, arsenic treatment decreased Bcl-2 and Bcl-xl protein expression and increased Bax, Bad and Caspase 3 protein expression compared with the control, but Pts pretreatment dramatically blocked these changes in a dose-dependent manner (*P* ≤ 0.01, as shown in Figure 5B–D).

### Pts Induced Nrf2 Translocation and Antioxidant Enzyme Expression in HaCaT Cells

**FIGURE 6:**
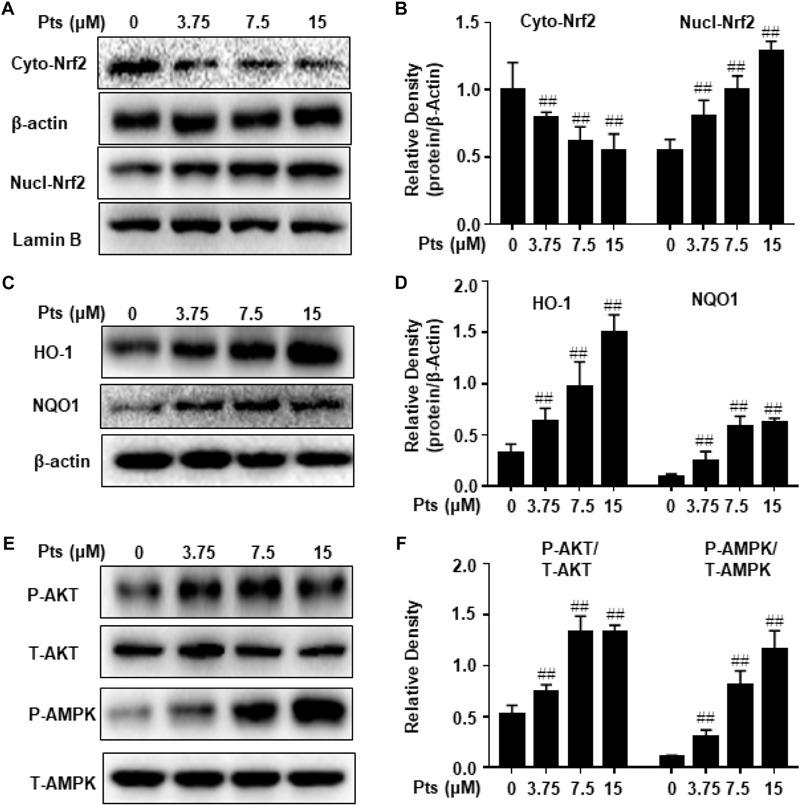
Effect of Pts on AMPK and AKT phosphorylation, Nrf2 translocation and antioxidant enzyme expression. HaCaT cells were plated in six-well culture plates and treated with Pts (3.75, 7.5, and 15 μM) for 24 h. Nuclear and cytoplasmic extracts from cells were prepared for detecting Nrf2 in panels **(A,B)** and total protein extracts from cells were prepared for detecting HO-1, NQO1, AMPK and AKT in panels **(C–F)**. The results are expressed as the means ± S.E.M. of three independent experiments. ^##^*P* ≤ 0.01 versus the control group.

NF-E2 p45-related factor 2 plays an imperative role in cellular redox homeostasis and actively regulates the expression of genes encoding antioxidant and phase 2 drug-metabolizing enzymes, such as HO-1, NQO-1, GCLC, and GCLM. As shown in Figure 6A,B, Pts treatment significantly increased the translocation of Nrf2 from cytoplasm to nucleus in a dose-dependent manner in HaCaT cells. Moreover, we quantified the expression of antioxidant enzymes and demonstrated that Pts upregulated HO-1 and NQO-1 expression in a dose-dependent manner (*P* ≤ 0.01, as shown in Figure 6C,D), but had no effect on GCLC and GCLM (data not shown). Pts has been shown to reduce acetaminophen-induced liver injury by activating the Nrf2 antioxidant defense system via the AMPK/Akt/GSK3β pathway (Fan et al., 2018). Thus, we aimed to examine the effect of Pts on AMPK and AKT phosphorylation. As shown in Figure 6E,F, treatment of cells with Pts markedly increased AMPK and AKT phosphorylation in a dose-dependent manner.

### Pts-Mediated Protective Effects on NaAsO_2_-Induced Cytotoxicity Are Dependent on Nrf2 Activation

**FIGURE 7:**
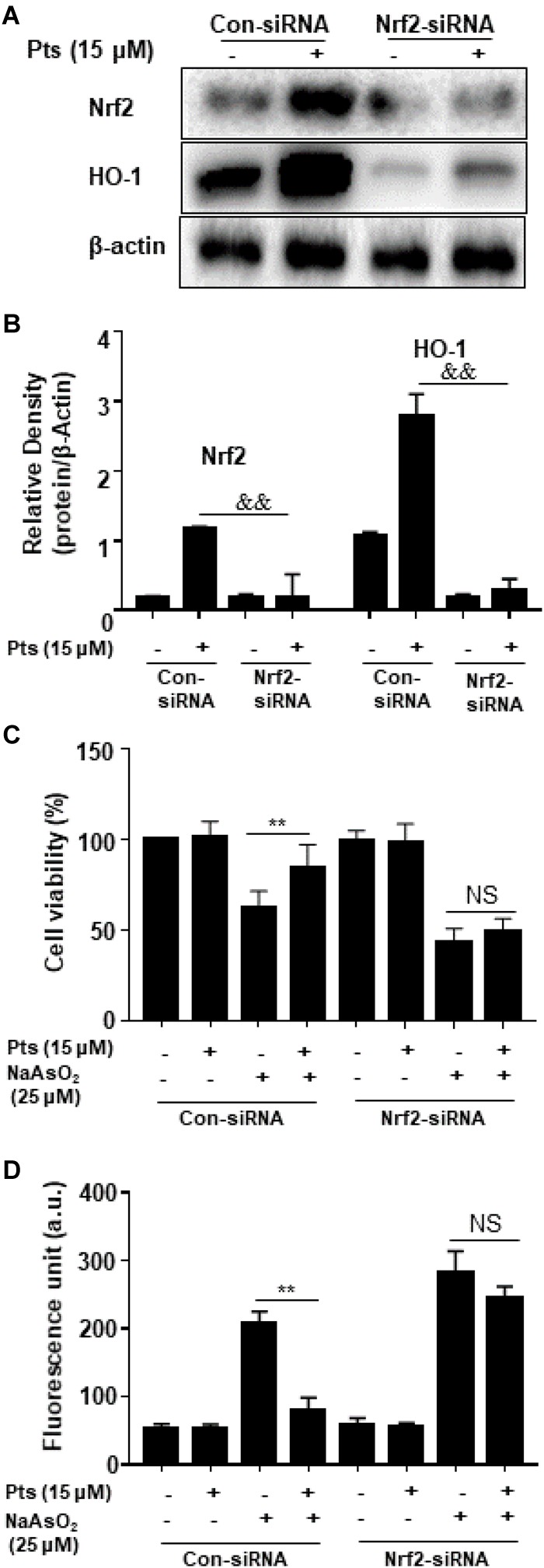
Effects of Nrf2-siRNA transfection on Pts-induced Nrf2 and HO-1 protein expression and protection on NaAsO_2_-induced cytotoxicity. **(A,B)** Nrf2 mediates Pts-induced HO-1 protein expression. Nrf2-siRNA or Nrf2-negative control siRNA were transfected into cells for 24 h and then were treated with Pts (15 μM) for 24 h. **(C)** Nrf2 mediates Pts-induced protection against NaAsO_2_-induced cytotoxicity. Cells were transfected with either control-siRNA or Nrf2-siRNA for 24 h, and then were left untreated or co-treated NaAsO_2_ (25 μM) with or without Pts (15 μM) for 24 h. Cell viability was measured using the MTT assay. **(D)** Nrf2 mediates Pts-induced protection against NaAsO_2_-induced oxidative stress in HaCaT cells. Cells were transfected with either control-siRNA or Nrf2-siRNA for 24 h, and then were left untreated or co-treated NaAsO_2_ (25 μM) with or without Pts (15 μM) for 24 h. ROS generation was measured by the DCFH-DA method using a multi-detection reader. The results are expressed as the means ± S.E.M. of three independent experiments. ^∗∗^ *P* ≤ 0.01 versus the NaAsO_2_ group.

We proposed that the protective effect of Pts on NaAsO_2_ cytotoxicity of human HaCaT cells is due to the activation of Nrf2. In our study, we used Nrf2 siRNA to confirm the effect of Pts on antioxidant enzymes and NaAsO_2_-induced cytotoxicity. HaCaT cells were transiently transfected with control or Nrf2 siRNA, and Nrf2 and HO-1 protein expression was detected by western blot analysis. Figure 7A,B demonstrate that Nrf2 siRNA can inhibit Nrf2 and HO-1 protein expression. Furthermore, our results showed that the protective effects of Pts on NaAsO_2_-induced cytotoxicity and ROS generation were remarkably attenuated in Nrf2 siRNA transfected cells (*P* ≤ 0.01, as shown in Figure 7C,D).

## Discussion

Pterostilbene has been shown to exert a wide range of biological effects on human health, including antioxidant properties. Pts has been used for the chemoprevention and treatment of various skin diseases, such as skin tumorigenesis and wound healing (Singh et al., 2017; Yang et al., 2017). It has been shown that pterostilbene activates Nrf2 in human keratinocytes to protect against UVB-induced photo damage (Singh et al., 2017). Skin pigmentation and keratosis have long been considered as the most common health problems associated with exposure to arsenic contaminated drinking water (Majumdar and Guha Mazumder, 2012). In this study, our data confirm the hypothesis that pterostilbene plays a vital role in protecting against NaAsO_2_-induced toxicity via activating the Nrf2 pathway.

Oxidative stress is pivotal in apoptosis and cell death in arsenic toxicology, which is caused by an imbalance in reactive species, including ROS, and the intracellular antioxidant defense system in cells. Pterostilbene has been reported to inhibit ROS production and prevent continued oxidative stress (He et al., 2018). Our results here suggested that arsenic-induced cytotoxicity were significantly reversed by Pts treatment. Moreover, Pts treatment significantly suppressed oxidative stress markers, such as the depletion of the antioxidant enzyme SOD and augmented ROS and MDA levels, which were caused by NaAsO_2_. In addition, mitochondria is essential for the normal maintenance of the physiological function of MMP, but the accumulation of ROS leads to mitochondrial dysfunction, which promotes the release of cytochrome c and cell apoptosis (Lv et al., 2017b). Our data suggested that Pts can reverse NaAsO_2_-induced MMP collapse and mitochondrial cytochrome c translocation, hence suggesting that Pts can attenuate NaAsO_2_-induced mitochondrial damage and prevent further apoptosis in HaCaT cells.

Apoptosis can be initiated by stressful conditions, resulting in serious health problems and fatality. Various models show that arsenic is a strong inducer of apoptosis by overgenerating ROS, decreasing mitochondrial membrane potential, and increasing the DNA fragmentation in immune cells (Xu et al., 2015). In the present study, we found that NaAsO_2_ caused excessive ROS production and eventually promoted apoptosis in HaCaT cells. Pts pretreatment ameliorated NaAsO_2_-induced apoptosis by decreasing the number of apoptotic cells and restoring the apoptotic morphological markers of chromatin condensation, nucleolus pyknosis, and apoptotic bodies in HaCaT cells. The Bcl-2 family of proteins includes the anti-apoptotic Bcl-2 subfamily, and the pro-apoptotic Bax subfamily and the Bik family, and the Bax/Bcl2 ratio plays a key role in cell death and survival. Previous findings indicate that Pts significantly decreases the incidence of apoptosis and the Bax/Bcl2 ratio during skeletal muscle IR injury (Cheng et al., 2016). Our current results showed that NaAsO_2_ exposure induced the downregulation of Bcl-2 and Bcl-xl protein and the upregulation of Bax, Bad and Caspase 3 proteins, which could be reversed by Pts treatment. Taken together, our results suggest that not only the regulation of mitochondrial dysfunction, but also the inhibition of the apoptotic process may be involved in the cytoprotective effects of the antioxidant Pts.

NF-E2 p45-related factor 2, which directly regulates many antioxidant and detoxification enzyme genes, plays a vital role in cellular redox homeostasis and antioxidant response. Previous studies have shown that the steady depletion of Nrf2 by shRNA makes human HaCaT cells more sensitive to iAs3+ induced cell death, suggesting that Nrf2 activators may be used in therapeutic and dietary interventions against the side effects of arsenic (Zhao et al., 2012). Pts, as an effective inducer of Nrf2, has been shown to reduce acetaminophen-induced liver injury by activating the Nrf2 antioxidant defense system via the AMPK/Akt/GSK3β pathway (Fan et al., 2018). In this study, Pts was shown to induce AMPK and AKT phosphorylation, translocation of Nrf2 from the cytoplasm to the nucleus, upregulating Nrf2 downstream expression of NQO1 and HO-1 expression. These investigations indicated that Pts possesses antioxidant activity and further protected against NaAsO_2_-induced toxicity in HaCaT cells, and that these effects are attenuated in Nrf2-knockdown cells, suggesting that the upregulation of Nrf2-mediated expression of antioxidant genes induced by Pts protects against NaAsO_2_-induced toxicity.

In conclusion, the present study demonstrated the protective effects of Pts against NaAsO_2_-induced cytotoxicity by regulating oxidative stress markers and mitochondrial dysfunction in HaCaT cells. Pts treatment provided protection from apoptosis by increasing Bcl-2 and decreasing Bax protein expression. In addition, Pts could also induce a Nrf2-dependent antioxidant response, which is likely connected to the observed increased cell survival. Our data therefore suggest that Pts, a Nrf2 inducer, represents a novel therapeutic and dietary candidate in the treatment of arsenic-induced skin hyperkeratosis and carcinogenesis. The protective effects of Pts on arsenic-induced skin damage should be further studied in animal models.

## Author Contributions

JZ wrote the manuscript and performed the experiments. XC, XM, QY, YC, and YZ performed some experiments, analyzed the data, and revised the manuscript. SL contributed to design the experiments and edited the manuscript.

## Conflict of Interest Statement

The authors declare that the research was conducted in the absence of any commercial or financial relationships that could be construed as a potential conflict of interest.
